# Selection and evaluation of preoperative systemic inflammatory response biomarkers model prior to cytoreductive nephrectomy using a machine-learning approach

**DOI:** 10.1007/s00345-021-03844-w

**Published:** 2021-10-20

**Authors:** Ekaterina Laukhtina, Victor M. Schuettfort, David D’Andrea, Benjamin Pradere, Fahad Quhal, Keiichiro Mori, Reza Sari Motlagh, Hadi Mostafaei, Satoshi Katayama, Nico C. Grossmann, Pawel Rajwa, Pierre I. Karakiewicz, Manuela Schmidinger, Harun Fajkovic, Dmitry Enikeev, Shahrokh F. Shariat

**Affiliations:** 1 Department of Urology, Comprehensive Cancer Center, Vienna General Hospital, Medical University of Vienna, Währinger Gürtel 18-20, 1090 Vienna, Austria; 2 Institute for Urology and Reproductive Health, Sechenov University, Moscow, Russia; 3 Department of Urology, University Medical Center Hamburg-Eppendorf, Hamburg, Germany; 4 Department of Urology, King Fahad Specialist Hospital, Dammam, Saudi Arabia; 5 Department of Urology, The Jikei University School of Medicine, Tokyo, Japan; 6 Men’s Health and Reproductive Health Research Center, Shahid Beheshti University of Medical Sciences, Tehran, Iran; 7 Research Center for Evidence Based Medicine, Tabriz University of Medical Sciences, Tabriz, Iran; 8 Department of Urology, Okayama University Graduate School of Medicine, Dentistry and Pharmaceutical Sciences, Okayama, Japan; 9 Department of Urology, University Hospital Zurich, Zurich, Switzerland; 10 Department of Urology, Medical University of Silesia, Zabrze, Poland; 11 Cancer Prognostics and Health Outcomes Unit, Division of Urology, University of Montreal Health Center, Montreal, QC Canada; 12 Karl Landsteiner Institute of Urology and Andrology, Vienna, Austria; 13 Department of Urology, Weill Cornell Medical College, New York, NY USA; 14 Department of Urology, University of Texas Southwestern, Dallas, TX USA; 15 Department of Urology, Second Faculty of Medicine, Charles University, Prague, Czech Republic; 16 Division of Urology, Department of Special Surgery, Jordan University Hospital, The University of Jordan, Amman, Jordan

**Keywords:** mRCC, Cytoreductive nephrectomy, CSS, AGR, DRR, SII

## Abstract

**Introduction:**

This study aimed to determine the prognostic value of a panel of SIR-biomarkers, relative to standard clinicopathological variables, to improve mRCC patient selection for cytoreductive nephrectomy (CN).

**Material and methods:**

A panel of preoperative SIR-biomarkers, including the albumin–globulin ratio (AGR), De Ritis ratio (DRR), and systemic immune-inflammation index (SII), was assessed in 613 patients treated with CN for mRCC. Patients were randomly divided into training and testing cohorts (65/35%). A machine learning-based variable selection approach (LASSO regression) was used for the fitting of the most informative, yet parsimonious multivariable models with respect to prognosis of cancer-specific survival (CSS). The discriminatory ability of the model was quantified using the C-index. After validation and calibration of the model, a nomogram was created, and decision curve analysis (DCA) was used to evaluate the clinical net benefit.

**Results:**

SIR-biomarkers were selected by the machine-learning process to be of high discriminatory power during the fitting of the model. Low AGR remained significantly associated with CSS in both training (HR 1.40, 95% CI 1.07–1.82, *p* = 0.01) and testing (HR 1.78, 95% CI 1.26–2.51, *p* = 0.01) cohorts. High levels of SII (HR 1.51, 95% CI 1.10–2.08, *p* = 0.01) and DRR (HR 1.41, 95% CI 1.01–1.96, *p* = 0.04) were associated with CSS only in the testing cohort. The exclusion of the SIR-biomarkers for the prognosis of CSS did not result in a significant decrease in C-index (− 0.9%) for the training cohort, while the exclusion of SIR-biomarkers led to a reduction in C-index in the testing cohort (− 5.8%). However, SIR-biomarkers only marginally increased the discriminatory ability of the respective model in comparison to the standard model.

**Conclusion:**

Despite the high discriminatory ability during the fitting of the model with machine-learning approach, the panel of readily available blood-based SIR-biomarkers failed to add a clinical benefit beyond the standard model.

**Supplementary Information:**

The online version contains supplementary material available at 10.1007/s00345-021-03844-w.

## Introduction

Cytoreductive nephrectomy (CN) with systemic therapy continues to be part of the treatment pathway in patients with metastatic renal cell carcinoma (mRCC) [1]. The role of CN has become increasingly controversial. Some studies have reported no difference in survival outcomes in the overall population of patients treated with or without CN before systemic therapy, while surgery was associated with improved survival only in specific patient subgroups [2]. Currently, to stratify mRCC patients and determine optimal therapeutic strategies, clinicians use the Memorial Sloan-Kettering Cancer Center (MSKCC, also known as Motzer score) [3] and the International metastatic renal cell carcinoma Database Consortium (IMDC, also known as Heng score) [4] risk models. However, significant intra-group heterogeneity exists among patients stratified according to MSKCC or IMDC categories. In consequence, an optimal patient selection for CN remains challenging. Accurate identification of patients who would benefit from CN for mRCC is an unmet clinical need.

Several blood-based systemic inflammatory response (SIR) biomarkers have been shown to have a high predictive value in various urological malignancies. SIR-biomarkers such as the albumin–globulin ratio (AGR) [5, 6], the De Ritis ratio (DRR) [7, 8], and the systemic immune-inflammation index (SII) [9, 10] have been evaluated to predict oncologic outcomes in RCC. Previous studies have already suggested that low AGR [11] as well as high DRR [12, 13] and SII [14, 15] could be potential biomarkers to predict worse overall survival (OS) and cancer-specific survival (CSS) in patients treated with CN for mRCC. Despite promising results, all single biomarkers have failed to provide a meaningful improvement to the discriminatory ability of standard models. A combination of complementary and independent biomarkers is more likely to capture a higher predictive value than any single biomarkers. We hypothesized that a panel of readily available blood-based SIR-biomarkers could improve outcome prediction in patients treated with CN for mRCC. Moreover, the use of a machine-learning-based variable selection approach could help determine the most effective predictors and create the most informative, yet parsimonious model with respect to clinically important outcome parameters.

This study aimed to select the most valuable predictors with respect to CSS using a machine-learning-based approach as well as determine the prognostic value of a panel of SIR-biomarkers relative to standard clinicopathological variables to improve mRCC patient selection for CN.

## Material and methods

### Study design

We retrospectively reviewed our established international multicenter database to identify mRCC patients treated with CN at tertiary centers in the USA and Europe. We excluded patients with other malignant primary tumors. However, concomitant hematologic or liver diseases, chronic inflammatory disease including autoimmune disorder and infection within the last 12 months were not excluded. The study was approved by ethics institutional committees at all participating institutions.

### Management

Dedicated uropathologists assigned pathologic stage according to the 2010 American Joint Committee on Cancer (AJCC) tumor, node and metastasis (TNM) staging system. All pathology reports from prior to 2010 were reviewed according to 2010 criteria. Patients were stratified according to the International Metastatic renal cell carcinoma Database Consortium (IMDC) [4].

All laboratory tests were done within 1 month prior to the CN. The serum AGR value was calculated as baseline serum albumin to baseline total protein–baseline serum albumin ratio. The DRR value was evaluated as the ratio of the serum activities of aspartate aminotransferase (AST) and alanine aminotransferase (ALT). The SII was based on neutrophil, lymphocyte, and platelet counts. The biomarkers and the respective cutoffs have previously been described in detail [11, 13]. The cutoffs of 1.43 for AGR, 1.2 for DRR, and 710 for SII were determined as having the maximum Youden index value. OS time was calculated from the date of CN to death or last follow-up. CSS time was calculated from the date of CN to death from disease or last follow-up.

### Statistical analysis

To simulate external validation and to perform a true performance assessment, we randomly divided patients into a training cohort (*n* = 400) and a testing cohort (*n* = 213) (65%/35%). Patients’ characteristics in the training and testing sets as well as the distribution of SIR-biomarkers were compared using the Pearson’s Chi-squared, Fisher’s exact, and Wilcoxon rank sum tests, as appropriate. We planned to use CSS as our primary end point according to the expected number of patients who died from cancer. Therefore, Cox model was fitted for the postoperative prognosis of CSS. The risk of survival was expressed as hazard ratios (HR) and 95% confidence intervals (95% CI).

The absolute shrinkage and selection operator (LASSO) approach and tenfold cross-validation were used for fitting of the most informative, yet parsimonious multivariable model with respect to prediction/prognosis of CSS. During the LASSO procedure, the absolute value of the regression coefficients of the assessed variables is continuously reduced through the use of a penalty. Using this penalty, which is the sum of the absolute size of the regression coefficients multiplied by a tuning parameter (lambda, *λ*), some coefficients are shrunk to zero. The corresponding variables hold little predictive value and can be neglected during the fitting of the model. The optimal weight of *λ* was determined by a tenfold cross-validation in the training set. For this purpose, the C-index across the cross-validation folds was calculated for increments of *λ*. The weight of *λ* that minimizes deviation in the cross-validation is given by *λ* min. However, the weight of *λ* is *λ*1.se, defined as the value of *λ* within one standard deviation of the minimum mean cross-validated error [16]. Variables whose LASSO coefficient was not equal to zero at *λ*1.se were extracted and used during the fitting of the prognostic model. This cross-validation process minimizes the risk of overfitting, and it is a way of assessing how a model will perform in an independent dataset. In summary, the LASSO procedure allows a machine-learning-based variable selection for the fitting of prognostic or predictive models. It has been suggested to be particularly well suited for variables that show high levels of multicollinearity, as to be expected for SIR-biomarkers [17, 18].

The selected variables were then used to fit the multivariable Cox model. The discrimination ability of this model was assessed by calculating the C-index (Harrell’s concordance index, an approximation of the AUC in censored data) for both the training and the testing cohorts. To assess the additional discriminatory power of the biomarkers, a reference model was fitted that did not include the previously selected SIR-biomarkers. Calibration plots graphically explored the association between predicted probabilities and the observed proportions. The goodness of fit of the Cox regression model was tested using the Grønnesby-and-Borgan test. Validation was performed using 200 bootstrap re-samples as a means of calculating the most unbiased predictive accuracy. Finally, the decision curve analysis (DCA) was used to evaluate the clinical net benefit of the model for both the training and testing cohorts [19, 20]. All reported *p* values were two-sided. Statistical significance was set at *p* < 0.05. All statistical analyses were performed using R (Version 4.0.3, Vienna, Austria, 2020).

## Results

Overall, 613 patients were included in the analysis. Patient characteristics were similar in both training (*n* = 400) and testing (*n* = 213) cohorts (Supplementary Table 1). The number of patients with a higher level of preoperative serum DRR was significantly higher in the testing cohort (42 vs. 33%, *p* = 0.04). At a median follow-up of 31 (IQR 16–58) months, a total of 472 (77%) patients died, and 99% of deaths were due to mRCC. Median CSS was 17 months (95% CI 15–23).

SIR-biomarkers were selected by the machine-learning process to be of high discriminatory power during the fitting of the model for prognosis of postoperative CSS (Supplementary Fig. 1, 2). Low AGR remained associated with worse CSS in both training (HR 1.40, 95% CI 1.07–1.82, *p* = 0.01) and testing (HR 1.78, 95% CI 1.26–2.51, *p* = 0.01) cohorts (Supplementary Table 2). High levels of SII (HR 1.51, 95% CI 1.10–2.08, *p* = 0.01) and DRR (HR 1.41, 95% CI 1.01–1.96, *p* = 0.04) were associated with worse CSS only in the testing cohort. In the testing cohort, a 200-fold bootstrap corrected C-index of 64.4% was found for the postoperative prognosis of CSS. The exclusion of the SIR-biomarkers for the prognosis of CSS did not result in a significant decrease in C-index (− 0.9%) for the training cohort, while the exclusion of SIR-biomarkers led to a reduction in C-index in the testing cohort (− 5.8%).

**Fig. 1:**
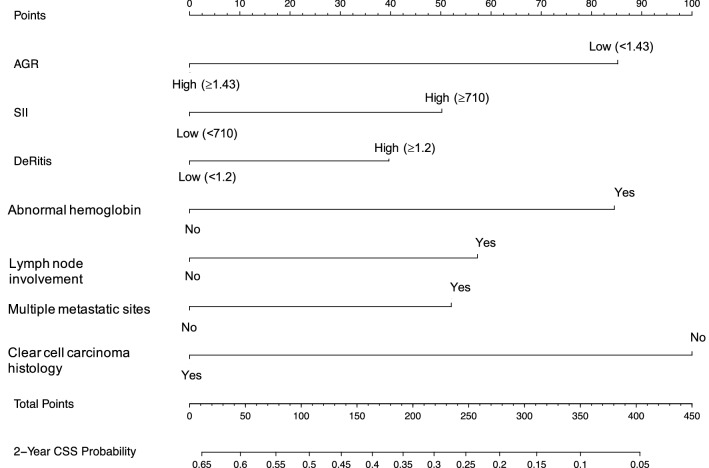
Postoperative nomogram predicting cancer-specific survival at 2 years based on the Cox regression model

**Fig. 2:**
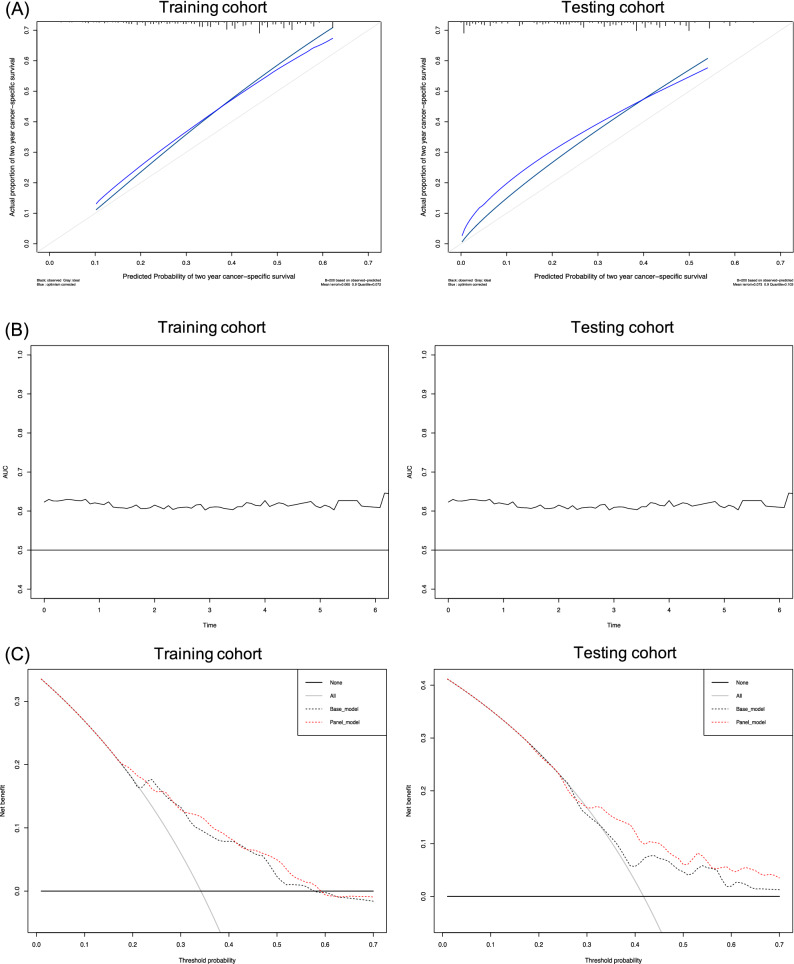
**A** Calibration plots of the postoperative nomogram predicting cancer-specific survival after cytoreductive nephrectomy. **B** Time-dependent area under the ROC curves for prediction of 2 year cancer-specific survival. **C** Decision curve analyses (DCA) for the evaluation of the clinical net benefit using the Cox model for prediction of 2 year cancer-specific survival

Assessment of the nomogram axes indicated that all of them demonstrate a wide range of predicted probabilities; AGR contributed to a high number of risk points (Fig. 1). The calibration plots showed that the model demonstrates a slight underprediction compared to actual outcome observation (Fig. 2A). In accordance with that, the goodness-of-fit tests were insignificant for all cohorts. For both cohorts, time-dependent AUC plots demonstrate a stable model performance over a period of 2 years (Fig. 2B). DCA showed that our model was associated with slight net benefit gain relative to the treat-all approach between a threshold probability of 40–50% in the testing cohort, while in the training cohort, the inclusion of the SIR-biomarkers did not improve the net benefit of the model (Fig. 2C).

## Discussion

Our approach of testing the incremental predictive accuracy of biomarkers compared to standard risk factors selected using a machine-learning-based approach could serve as a benchmark for evaluating novel biomarkers. Using a machine-learning-based approach, we were able to select the most valuable predictors of CSS in mRCC patients treated with CN. For prognosis of CSS, blood-based SIR-biomarkers were chosen for the fitting of the most accurate model.

Our analyses found that low AGR remained significantly associated with worse CSS in both training and testing cohorts, while high levels of SII and DRR were associated with worse CSS only in the testing cohort. Nevertheless, the SIR-biomarkers did not result in a significant decrease in C-index in the training cohort, while the exclusion of SIR-biomarkers led to a significant reduction in C-index in the testing cohort. In agreement with these findings, in a study of 146 mRCC patients treated with CN, Kalogirou et al. reported that preoperative C-reactive protein levels improved the accuracy of the nomogram aimed at identifying the candidates who are most likely to benefit from CN (accuracy 60.8 vs. 69.7%) [21]. Margulis et al. reported a preoperative nomogram, including serum albumin and serum lactate dehydrogenase, for prediction of CSS after CN resulting in a discrimination of 0.76 [22]. The recent studies also suggested the different SIR-biomarkers as a predictive value in mRCC patients treated with systemic therapy. Kim et al. reported that in mRCC patients treated with first-line targeted therapy, a new model that incorporated DRR and neutrophil-to-lymphocyte ratio (NLR) had significantly better predictive value for OS (C-index = 0.727) compared to both the IMDC and MSKCC risk models (C-index = 0.661 and 0.612, respectively) [23]. Xu et al. reported a superior discriminatory ability for OS among other SIR-biomarkers in patients with spinal RCC metastases treated with first-line targeted therapy; the SIR-markers were NLR and platelet–lymphocyte ratio (PLR) [24]. Ramsey et al. reported an inflammation‐based prognostic score predicting survival in mRCC patients regardless of treatment option; this score was independent of established scoring systems [25]. Despite the use of different SIR-biomarkers, the inherent heterogeneity and limitations of these and our studies, the cumulative evidence suggests that SIR-biomarkers hold promise to improve survival stratification beyond the current standard risk mRCC models. Cheap, reproducible, and readily available SIR-biomarkers can change the clinical decision-making process in mRCC patients treated with CN [26].

According to our results, the nomogram comprising the most valuable predictors of CSS had a stable performance with a slight underprediction. However, the accuracy of our models was similar for both the internal and the external validation cohorts; it was also comparable to some of the previously reported nomograms [21, 27]. Nevertheless, our nomogram failed to reach a clinically acceptable prognostic performance as its accuracy did not reach 75% on external validation [28]. Calibration and validation of predictive models or nomograms are paramount before their implementation into daily clinical practice. We, therefore, imitated external validation by splitting our patient population into training and testing cohorts. However, real external validation using separate cohorts from different centers is the best method to assess a model’s accuracy.

We believe that conventional multivariable analyses and the change in C-index that quantify the ability of the model to discriminate between patients with and those without the outcome of interest are not sufficient to demonstrate that a panel has a clinical benefit [19]. Indeed, to explore this, we used DCA, a method that combines simplicity with efficient computations [19]. Comparing our predictive model to a similar reference model that excluded any SIR-biomarkers revealed that the addition of the selected SIR-biomarkers only marginally improved the net benefit of the standard model by what is estimated to be a clinically non-significant margin. It should be highlighted that systemic treatment with TKI in our study was used according to recommendations at the time prior to the advent of immunotherapy. Ideally, the contemporary predictive value of SIR-biomarkers should be assessed in the era of immunotherapy. Due to the strong association with immunoinflammatory reactions, SIR-biomarkers might significantly improve the net benefit of the standard model for the prediction of oncologic outcomes in mRCC patients treated with immune checkpoint inhibitors.

Our study is not devoid of limitations. The main limitation of the study was its retrospective and multicenter design, which may result in a lack of standardized laboratory, pathologic, surgical, and treatment approaches that could confound the results. We did not adjust our model for postoperative treatment strategies because of the heterogeneity of this information. However, in our study, TKI therapy used according to recommendations at the time of data collection is very unlikely to alter OS or CSS. Another limitation of our study is the fact that SIR-biomarkers might have been biased by the presence of undetected liver, hematologic, or immunologic diseases. Additionally, SIR-biomarkers were assessed preoperatively at a single time point. SIR-biomarkers variability over time, in response to therapy and its relationship to the oncological prognosis of mRCC patients, have not been tested. Despite all these limitations, we presented the largest series investigating the association of preoperative SIR-biomarkers with oncologic outcomes in mRCC patients treated with CN. Further well-designed large-scale studies should be conducted to validate our promising results.

## Conclusion

Despite the high discriminatory ability during the fitting of the model with machine-learning approach, the panel of readily available blood-based SIR-biomarkers failed to add a clinical benefit beyond that afforded by the standard model. Novel biomarkers are needed to improve outcome prognosis in this setting. This study could be the benchmark for further evaluation of blood-based SIR-biomarkers as prognostic biomarkers, the importance of which is increasing, especially in the era of immunotherapy.

## Supplementary Information

Below is the link to the electronic supplementary material.

Supplementary file1 (PDF 40 KB) Association of the preoperative blood-based systemic inflammatory response biomarkers with clinicopathologic characteristics in 613 patients treated with cytoreductive nephrectomy for metastatic renal cell carcinoma.

Supplementary file2 (DOCX 80 KB)

## References

[1] Ljungberg B (2020). Renal cell carcinoma EAU guidelines on renal cell carcinoma: 2020. Eur Urol.

[2] Janisch F (2020). The impact of cytoreductive nephrectomy on survival outcomes in patients treated with tyrosine kinase inhibitors for metastatic renal cell carcinoma in a real-world cohort. Urol Oncol Semin Orig Investig.

[3] Motzer RJ, Mazumdar M, Bacik J, Berg W, Amsterdam A, Ferrara J (1999). Survival and prognostic stratification of 670 patients with advanced renal cell carcinoma. J Clin Oncol.

[4] Heng DYC (2009). Prognostic factors for overall survival in patients with metastatic renal cell carcinoma treated with vascular endothelial growth factor-targeted agents: results from a large, multicenter study. J Clin Oncol.

[5] Chen Z (2017). Preoperative albumin to globulin ratio predicts survival in clear cell renal cell carcinoma patients. Oncotarget.

[6] He X (2017). Preoperative albumin to globulin ratio (AGR) as prognostic factor in renal cell carcinoma. J Cancer.

[7] Ikeda T (2020). The De Ritis (aspartate transaminase/alanine transaminase) ratio as a prognosticator in patients with end-stage renal disease–associated renal cell carcinoma. Clin Genitourin Cancer.

[8] Lee H, Lee SE, Byun SS, Kim HH, Kwak C, Hong SK (2017). De Ritis ratio (aspartate transaminase/alanine transaminase ratio) as a significant prognostic factor after surgical treatment in patients with clear-cell localized renal cell carcinoma: a propensity score-matched study. BJU Int.

[9] Lolli C (2016). Systemic immune-inflammation index predicts the clinical outcome in patients with metastatic renal cell cancer treated with sunitinib. Oncotarget.

[10] Chrom P, Zolnierek J, Bodnar L, Stec R, Szczylik C (2019). External validation of the systemic immune-inflammation index as a prognostic factor in metastatic renal cell carcinoma and its implementation within the international metastatic renal cell carcinoma database consortium model. Int J Clin Oncol.

[11] Laukhtina E (2020). Prognostic effect of preoperative serum albumin to globulin ratio in patients treated with cytoreductive nephrectomy for metastatic renal cell carcinoma. Transl Androl Urol.

[12] Ishihara H (2017). Evaluation of preoperative aspartate transaminase/alanine transaminase ratio as an independent predictive biomarker in patients with metastatic renal cell carcinoma undergoing cytoreductive nephrectomy: a propensity score matching study. Clin Genitourin Cancer.

[13] Laukhtina E (2020). Association of preoperative serum De Ritis ratio with oncological outcomes in patients treated with cytoreductive nephrectomy for metastatic renal cell carcinoma. Urol Oncol Semin Orig Investig.

[14] Barua SK (2019). Predictors of progression-free survival and overall survival in metastatic non-clear cell renal cell carcinoma: a single-center experience. World J Oncol.

[15] Fukuda H, Takagi T, Kondo T, Shimizu S, Tanabe K (2018). Predictive value of inflammation-based prognostic scores in patients with metastatic renal cell carcinoma treated with cytoreductive nephrectomy. Oncotarget.

[16] Krstajic D, Buturovic LJ, Leahy DE, Thomas S (2014). Cross-validation pitfalls when selecting and assessing regression and classification models. J Cheminform.

[17] Tibshirani R (2011). Regression shrinkage and selection via the lasso: a retrospective. J R Stat Soc Ser B Stat Methodol.

[18] Friedman J, Hastie T, Tibshirani R (2010). Regularization paths for generalized linear models via coordinate descent. J Stat Softw.

[19] Shariat SF (2010). Statistical consideration for clinical biomarker research in bladder cancer. Urol Oncol Semin Orig Investig.

[20] D’Andrea D (2019). Diagnostic accuracy, clinical utility and influence on decision-making of a methylation urine biomarker test in the surveillance of non-muscle-invasive bladder cancer. BJU Int.

[21] Kalogirou C (2017). Preoperative C-reactive protein values as a potential component in outcome prediction models of metastasized renal cell carcinoma patients receiving cytoreductive nephrectomy. Urol Int.

[22] Margulis V (2013). Development of accurate models for individualized prediction of survival after cytoreductive nephrectomy for metastatic renal cell carcinoma. Eur Urol.

[23] Kim SH, Park EY, Joo J, Chung J (2018). The De Ritis and neutrophil-to-lymphocyte ratios may aid in the risk assessment of patients with metastatic renal cell carcinoma. J Oncol.

[24] Xu K (2020). Prognostic significance of preoperative inflammatory biomarkers and traditional clinical parameters in patients with spinal metastasis from clear cell renal cell carcinoma: a retrospective study of 95 patients in a single center. Cancer Manag Res.

[25] Ramsey S, Lamb GWA, Aitchison M, Graham J, McMillan DC (2007). Evaluation of an inflammation-based prognostic score in patients with metastatic renal cancer. Cancer.

[26] Bensalah K, Montorsi F, Shariat SF (2007). Challenges of cancer biomarker profiling. Eur Urol.

[27] Manley BJ (2017). The difficulty in selecting patients for cytoreductive nephrectomy: an evaluation of previously described predictive models. Urol Oncol Semin Orig Investig.

[28] Bianco FJ (2006). Nomograms and medicine. Eur Urol.

