# Supplementary material for: Selection and evaluation of preoperative systemic inflammatory response biomarkers model prior to cytoreductive nephrectomy using a machine-learning approach
Source: World J Urol. 2021 Oct 20;40(3):747–54. doi: 10.1007/s00345-021-03844-w (PMC8948147; doi:10.1007/s00345-021-03844-w)
Supplement: Supplementary file 2 — Supplementary file2 (DOCX 80 KB) [file 345_2021_3844_MOESM2_ESM.docx]

**Supplementary Table 2. Multivariable Cox regression models for prediction cancer-specific survival in patients treated with cytoreductive nephrectomy for metastatic renal cell carcinoma. The Cox regression model was created using LASSO regression with 10-folds cross validation.**

|  | Training cohort | | | Testing cohort | | |
| --- | --- | --- | --- | --- | --- | --- |
|  | HR | 95% CI | p-value | HR | 95% CI | p-value |
| AGR < 1.43 | 1.40 | 1.07 - 1.82 | **0.01** | 1.78 | 1.26 - 2.51 | **<0.001** |
| SII ≥ 710 | 1.23 | 0.98 - 1.55 | 0.07 | 1.51 | 1.10 - 2.08 | **0.01** |
| DRR ≥ 1.2 | 1.21 | 0.96 - 1.52 | 0.1 | 1.41 | 1.01 - 1.96 | **0.04** |
| Age | 1.02 | 1.01 - 1.03 | **<0.001** | 1.00 | 0.99 - 1.02 | 0.91 |
| Abnormal hemoglobin | 1.59 | 1.26 - 2.01 | **<0.001** | 1.39 | 0.99 - 1.96 | 0.06 |
| Lymph node involvement | 1.49 | 1.09 - 1.95 | **0.01** | 1.22 | 0.79 - 1.88 | 0.36 |
| Multiple metastatic sites | 1.27 | 0.98 - 1.64 | 0.08 | 1.51 | 1.07 - 2.12 | **0.02** |
| Clear cell carcinoma histology | 0.52 | 0.38 - 0.73 | **<0.001** | 0.60 | 0.37 - 0.98 | **0.04** |
| 200-fold bootstrap corrected C-index | 0.664 | | | 0.644 | | |
| C-index with biomarkers | 0.663 | | | 0.645 | | |
| C-index without biomarkers | 0.654 | | | 0.597 | | |
| HR = Hazard Ratio, CI = Confidence Interval | | | | | | |

Bold p values are considered statistically significant (p value < 0.05).

AGR - albumin-globulin ratio; BMI - body mass index; DRR - De Ritis ratio; ECOG - Eastern Cooperative Oncology Group performance status; IMDC - International Metastatic RCC Database Consortium Risk Model; SII - systemic immune-inflammation index.

**Supplementary** **Figure 1. Lasso Coefficient profiles of all prognosis related variables in the training cohort (n=400).**

List of variables used: AGR, DRR, SII, age, gender, BMI, ECOG, IMDC, Karnofsky performance status, time from diagnosis to nephrectomy less than 12 months, abnormal level of hemoglobin, calcium, neutrophils, platelet, metastases sites (lung, bone, liver, brain, lymph nodes, adrenal gland) multiple metastatic sites, systemic therapy before CN, clear cell carcinoma histology, sarcomatoid features.

**Supplementary Figure 2. 10-fold cross validation for tuning parameter selection in the least absolute shrinkage and selection operator (LASSO) Model.**

Red dotted line: 10-fold cross validation curve along the λ sequence (error bars represent the upper and lower standard deviation). Two selected λ‘s are indicated by the vertical dotted lines (left: minimum λ = the value of λ that gives minimum mean cross-validated error. Right: 1.se. λ = cross validated error within one standard deviation of the minimum).
