# Supplementary material for: Selection and evaluation of preoperative systemic inflammatory response biomarkers model prior to cytoreductive nephrectomy using a machine-learning approach
Source: World J Urol. 2021 Oct 20;40(3):747–54. doi: 10.1007/s00345-021-03844-w (PMC8948147; doi:10.1007/s00345-021-03844-w)
Supplement: Supplementary file 1 — Supplementary file1 (PDF 40 KB) Association of the preoperative blood-based systemic inflammatory response biomarkers with clinicopathologic characteristics in 613 patients treated with cytoreductive nephrectomy for metastatic renal cell carcinoma. [file 345_2021_3844_MOESM1_ESM.pdf]

Characteristic	Overall	Cohort stratification			AGR			DRR			SII		
	N = 613	Testing cohort, N = 400	Training cohort N = 213	p-value	AGR≥ 1.43 N = 454	AGR<1.43 N = 159	p-value	DRR<1.2 N = 374	DRR≥1.2 N = 239	p-value	SII<710 N = 315	SII≥710 N = 298	p-value
Age, median (IQR)	57 (50, 64)	57 (50, 64)	57 (49, 64)	>0.9	57 (49, 64)	58 (51, 65)	0.4	57 (50, 64)	57 (50, 64)	0.6	57 (51, 64)	56 (49, 64)	0.5
Gender, n (%)				0.3			0.11			0.11			0.7
Male	428 (70%)	285 (71%)	143 (67%)		325 (72%)	103 (65%)		270 (72%)	158 (66%)		218 (69%)	210 (70%)	
Female	185 (30%)	115 (29%)	70 (33%)		129 (28%)	56 (35%)		104 (28%)	81 (34%)		97 (31%)	88 (30%)	
BMI, median (IQR)	27.5 (24.5, 30.0)	27.7 (24.6, 30.0)	27.2 (24.4, 30.0)	0.7	27.5 (24.6, 30.0)	28.0 (24.2, 30.2)	>0.9	28.0 (24.8, 30.5)	27.0 (24.2, 29.1)	**0.013**	27.1 (24.1, 30.0)	28.0 (24.8, 30.0)	0.10
BMI groups, n (%)				0.6			0.7			0.12			0.090
BMI ≥25	178 (29%)	113 (28%)	65 (31%)		130 (29%)	48 (30%)		100 (27%)	78 (33%)		101 (32%)	77 (26%)	
BMI<25	435 (71%)	287 (72%)	148 (69%)		324 (71%)	111 (70%)		274 (73%)	161 (67%)		214 (68%)	221 (74%)	
ECOG before nephrectomy, n (%)				0.7			0.9			>0.9			0.4
0	410 (67%)	271 (68%)	139 (65%)		305 (67%)	105 (66%)		248 (66%)	162 (68%)		204 (65%)	206 (69%)	
1	186 (30%)	117 (29%)	69 (32%)		137 (30%)	49 (31%)		115 (31%)	71 (30%)		103 (33%)	83 (28%)	
2	17 (2.8%)	12 (3.0%)	5 (2.3%)		12 (2.6%)	5 (3.1%)		11 (2.9%)	6 (2.5%)		8 (2.5%)	9 (3.0%)	
Prognostic groups according to IMDC criteria, n (%)				0.6			0.2			0.062			0.5
Favorable	186 (30%)	125 (31%)	61 (29%)		146 (32%)	40 (25%)		109 (29%)	77 (32%)		89 (28%)	97 (33%)	
Intermediate	343 (56%)	224 (56%)	119 (56%)		251 (55%)	92 (58%)		204 (55%)	139 (58%)		181 (57%)	162 (54%)	
Poor	84 (14%)	51 (13%)	33 (15%)		57 (13%)	27 (17%)		61 (16%)	23 (9.6%)		45 (14%)	39 (13%)	
Karnofsky Performance status				0.6			0.8			0.8			0.7
≥80%	596 (97%)	388 (97%)	208 (98%)		442 (97%)	154 (97%)		363 (97%)	233 (97%)		307 (97%)	289 (97%)	
<80%	17 (2.8%)	12 (3.0%)	5 (2.3%)		12 (2.6%)	5 (3.1%)		11 (2.9%)	6 (2.5%)		8 (2.5%)	9 (3.0%)	
Hemoglobin g/dL, median (IQR)	11.90 (10.50, 13.50)	11.90 (10.60, 13.60)	11.90 (10.40, 13.30)	0.6	12.00 (10.70, 13.60)	11.70 (10.05, 13.20)	0.081	11.90 (10.70, 13.60)	11.90 (10.30, 13.40)	0.5	11.90 (10.40, 13.50)	12.00 (10.60, 13.50)	0.4
Abnormal hemoglobin, n (%)				0.9			0.11			>0.9			0.3
≥LLN	299 (49%)	196 (49%)	103 (48%)		230 (51%)	69 (43%)		182 (49%)	117 (49%)		147 (47%)	152 (51%)	
<LLN	314 (51%)	204 (51%)	110 (52%)		224 (49%)	90 (57%)		192 (51%)	122 (51%)		168 (53%)	146 (49%)	
Calcium, mg/dL, median (IQR)	9.30 (8.90, 9.70)	9.30 (8.80, 9.62)	9.30 (9.00, 9.80)	0.11	9.30 (8.90, 9.70)	9.30 (8.85, 9.70)	0.9	9.30 (8.90, 9.70)	9.30 (8.90, 9.70)	>0.9	9.30 (8.90, 9.70)	9.30 (8.90, 9.70)	0.9
Abnormal calcium, n (%)				0.2			0.5			0.14			0.4
≤ULN	556 (91%)	367 (92%)	189 (89%)		414 (91%)	142 (89%)		334 (89%)	222 (93%)		289 (92%)	267 (90%)	
>ULN	57 (9.3%)	33 (8.2%)	24 (11%)		40 (8.8%)	17 (11%)		40 (11%)	17 (7.1%)		26 (8.3%)	31 (10%)	
Neutrophils, 109/L, median (IQR)	5.50 (4.20, 7.00)	5.50 (4.10, 7.00)	5.40 (4.30, 6.70)	>0.9	5.40 (4.20, 6.80)	5.60 (4.40, 7.20)	0.3	5.60 (4.40, 7.10)	5.20 (4.10, 6.60)	0.056	5.50 (4.40, 7.00)	5.45 (4.10, 6.90)	0.5
Abnormal neutrophils, n (%)				>0.9			0.3			0.15			0.7
≤ULN	463 (76%)	302 (76%)	161 (76%)		348 (77%)	115 (72%)		275 (74%)	188 (79%)		236 (75%)	227 (76%)	
>ULN	150 (24%)	98 (24%)	52 (24%)		106 (23%)	44 (28%)		99 (26%)	51 (21%)		79 (25%)	71 (24%)	
Platelet, 109/L, median (IQR)	312 (240, 410)	306 (234, 406)	320 (248, 421)	0.2	305 (240, 399)	333 (236, 445)	0.13	324 (239, 410)	302 (243, 414)	0.7	323 (242, 420)	304 (236, 400)	0.3
Abnormal platelet, n (%)				0.4			**0.011**			>0.9			0.13
≤ULN	448 (73%)	297 (74%)	151 (71%)		344 (76%)	104 (65%)		273 (73%)	175 (73%)		222 (70%)	226 (76%)	
>ULN	165 (27%)	103 (26%)	62 (29%)		110 (24%)	55 (35%)		101 (27%)	64 (27%)		93 (30%)	72 (24%)	
Time from diagnosis to nephrectomy				0.2			0.13			0.4			0.7
Less than 12 months	584 (95%)	384 (96%)	200 (94%)		436 (96%)	148 (93%)		354 (95%)	230 (96%)		301 (96%)	283 (95%)	
More than 12 months	29 (4.7%)	16 (4.0%)	13 (6.1%)		18 (4.0%)	11 (6.9%)		20 (5.3%)	9 (3.8%)		14 (4.4%)	15 (5.0%)	
Largest metastasis size, cm, median (IQR)	2.00 (2.00, 3.00)	2.00 (2.00, 3.00)	2.00 (2.00, 3.00)	>0.9	2.00 (2.00, 3.00)	2.00 (2.00, 3.00)	>0.9	2.00 (2.00, 3.00)	2.00 (2.00, 3.00)	0.4	2.00 (2.00, 3.00)	2.00 (2.00, 3.00)	0.6
Number of metastasis sites, n (%)				0.4			0.3			0.5			0.2
Single	370 (60%)	246 (62%)	124 (58%)		280 (62%)	90 (57%)		230 (61%)	140 (59%)		198 (63%)	172 (58%)	
Multiple	243 (40%)	154 (38%)	89 (42%)		174 (38%)	69 (43%)		144 (39%)	99 (41%)		117 (37%)	126 (42%)	
Site of metastases, n (%)													
Lung	415 (68%)	272 (68%)	143 (67%)	0.8	303 (67%)	112 (70%)	0.4	255 (68%)	160 (67%)	0.7	214 (68%)	201 (67%)	0.9
Bones	181 (30%)	114 (28%)	67 (31%)	0.4	138 (30%)	43 (27%)	0.4	121 (32%)	60 (25%)	0.055	94 (30%)	87 (29%)	0.9
Liver	41 (6.7%)	26 (6.5%)	15 (7.0%)	0.8	24 (5.3%)	17 (11%)	**0.019**	14 (3.7%)	27 (11%)	**<0.001**	21 (6.7%)	20 (6.7%)	>0.9
Brain	20 (3.3%)	9 (2.2%)	11 (5.2%)	0.053	15 (3.3%)	5 (3.1%)	>0.9	17 (4.5%)	3 (1.3%)	**0.025**	9 (2.9%)	11 (3.7%)	0.6
Lymph nodes	138 (23%)	92 (23%)	46 (22%)	0.7	96 (21%)	42 (26%)	0.2	86 (23%)	52 (22%)	0.7	58 (18%)	80 (27%)	**0.012**
Adrenal glands	91 (15%)	64 (16%)	27 (13%)	0.3	64 (14%)	27 (17%)	0.4	50 (13%)	41 (17%)	0.2	42 (13%)	49 (16%)	0.3
Other	38 (6.2%)	22 (5.5%)	16 (7.5%)	0.3	32 (7.0%)	6 (3.8%)	0.14	19 (5.1%)	19 (7.9%)	0.2	20 (6.3%)	18 (6.0%)	0.9
Neoadjuvant therapy, n (%)				0.9			0.4			0.8			0.7
No	500 (82%)	327 (82%)	173 (81%)		374 (82%)	126 (79%)		304 (81%)	196 (82%)		255 (81%)	245 (82%)	
Yes	113 (18%)	73 (18%)	40 (19%)		80 (18%)	33 (21%)		70 (19%)	43 (18%)		60 (19%)	53 (18%)	
Lymphadenectomy, n (%)	252 (41%)	165 (41%)	87 (41%)	>0.9	195 (43%)	57 (36%)	0.12	154 (41%)	98 (41%)	>0.9	126 (40%)	126 (42%)	0.6
Histology, n (%)				>0.9			0.4			0.4			0.2
Clear cell carcinoma	529 (86%)	345 (86%)	184 (86%)		395 (87%)	134 (84%)		319 (85%)	210 (88%)		277 (88%)	252 (85%)	
Other	84 (14%)	55 (14%)	29 (14%)		59 (13%)	25 (16%)		55 (15%)	29 (12%)		38 (12%)	46 (15%)	
Sarcomatoid features, n (%)				0.4			0.3			0.8			0.7
No	502 (82%)	331 (83%)	171 (80%)		376 (83%)	126 (79%)		305 (82%)	197 (82%)		260 (83%)	242 (81%)	
Yes	111 (18%)	69 (17%)	42 (20%)		78 (17%)	33 (21%)		69 (18%)	42 (18%)		55 (17%)	56 (19%)	
Grade, n (%)				0.5			0.3			0.052			0.7
G1/G2	65 (11%)	47 (12%)	18 (8%)		52 (11%)	13 (8%)		34 (9%)	31 (13%)		29 (9%)	36 (12%)	
G3/G4	548 (89%)	353 (88%)	195 (92%)		402 (89%)	146 (92%)		340 (91%)	208 (87%)		286 (91%)	262 (88%)	
Adjuvant therapy, n (%)				0.093			0.2			**<0.001**			0.7
No	132 (22%)	78 (20%)	54 (25%)		103 (23%)	29 (18%)		61 (16%)	71 (30%)		66 (21%)	66 (22%)	
Yes	481 (78%)	322 (80%)	159 (75%)		351 (77%)	130 (82%)		313 (84%)	168 (70%)		249 (79%)	232 (78%)	
AGR				0.059						0.9			0.2
AGR≥ 1.43	454 (74%)	306 (76%)	148 (69%)					276 (74%)	178 (74%)		226 (72%)	228 (77%)	
AGR<1.43	159 (26%)	94 (24%)	65 (31%)					98 (26%)	61 (26%)		89 (28%)	70 (23%)	
DRR				**0.036**			0.9						0.073
DRR<1.2	374 (61%)	232 (58%)	142 (67%)		276 (61%)	98 (62%)					203 (64%)	171 (57%)	
DRR≥1.2	239 (39%)	168 (42%)	71 (33%)		178 (39%)	61 (38%)					112 (36%)	127 (43%)	
SII				0.3			0.2			0.073			
SII<710	315 (51%)	200 (50%)	115 (54%)		226 (50%)	89 (56%)		203 (54%)	112 (47%)				
SII≥71	298 (49%)	200 (50%)	98 (46%)		228 (50%)	70 (44%)		171 (46%)	127 (53%)				

Statistics presented: n (%); Median (IQR)

Statistical tests performed: Pearson's Chi-squared test; Fisher's exact test; Wilcoxon rank sum test

AGR - albumin-globulin ratio; BMI - body mass index; DRR - De Ritis ratio; ECOG - Eastern Cooperative Oncology Group performance status; IMDC - International Metastatic RCC Database Consortium Risk Model; IQR - interquartile range; LLN – lower limit of normal; SII - systemic immune-inflammation index; ULN - upper limit of normal
